# Rapidly developable therapeutic-grade equine immunoglobulin against the SARS-CoV-2 infection in rhesus macaques

**DOI:** 10.1038/s41392-022-01095-8

**Published:** 2022-07-07

**Authors:** Xiaolei Liu, Yi Liu, Xuemin Jin, Zhanlong He, Zhen Huang, Shumin Sun, Yuwei Gao, Jingyu Li, Qin Ning, Zhongping Xie, Ningyi Jin, Mingyuan Liu

**Affiliations:** 1 State Key Laboratory for Zoonotic Diseases, Key Laboratory for Zoonosis Research of the Ministry of Education, Institute of Zoonosis, and College of Veterinary Medicine, Jilin University, 130062 Changchun, China; 2 Institute of Medical Biology, Chinese Academy of Medicine Sciences & Peking Union Medical College, Yunnan Key Laboratory of Vaccine Research and Development on Severe Infectious Diseases, 650118 Kunming, China; 3 Walvax Biotechnology Co., Ltd, 650106 Kunming, China; 4 College of Animal Science and Technology, Inner Mongolia University for Nationalities, 028000 Tongliao, China; 5 Changchun Veterinary Research Institute, Chinese Academy of Agricultural Sciences, Changchun, China; 6 Yuxi JOZO Biotechnology co., LTD, 653199 Yuxi, China; 7 National Medical Center for Major Public Health Events, State Key Laboratory for Zoonotic Diseases, Department and Institute of Infectious Disease, Tongji Hospital, Tongji Medical College, Huazhong University of Science and Technology, Wuhan, China

**Keywords:** Infection, Infectious diseases

**Dear Editor**,

The unprecedented COVID-19 pandemic caused by SARS-CoV-2 remains ongoing, but there is a lack of fully effective treatments. Convalescent plasma-derived hyperimmune globulins have been a safe and effective treatment but restricted by the difficulties in obtaining sufficient plasma with high antibody titers from a large number of recovered patients. Heterologous antibodies, particularly equine antibodies, have been widely used for decades as the therapeutics against some viral infections or as antivenoms.^1^ Equine antibodies could be rapidly developed and manufactured into therapeutic antibodies in large quantities under WHO standardized guidelines. Here we explored the development of equine antibody-derived F(ab′)_2_ as an option to treat COVID-19 by targeting the receptor-binding domain (RBD) of the viral spike protein that is essential for the viral entry into the host cells.^2^ We observed excellent neutralization titers of the F(ab′)_2_ in vitro and high potency against SARS-CoV-2 infection in the nonhuman primate rhesus macaques.

In this study, ten healthy horses (4–6-year old) were immunized with recombinant RBD (rRBD) with satisfactory binding activity to ACE2 (Supplementary Fig. S1a, b). Each horse was subcutaneously immunized with rRBD (based on GenBank No. 43740568) emulsified with Freund's adjuvants on days 0, 7, 14, 21, 28, and 42 (Fig. 1a). Serums were collected weekly (prior to injections on days when animals receiving immunization). Anti-RBD antibody started to elevate on day 14, rose to near the highest on day 21, and slightly increased more on days 35 and 42 (Supplementary Fig. S1c). In vitro anti-SARS-CoV-2 assay (KMS-1 isolate; genome sequence at GenBank: MT226610) for serums collected on days 21, 35, and 42, the 50% plaque reduction neutralization titers (PRNT_50_) reached 1/9000, 1/10,000 and 1/12,000, respectively (Fig. 1b). Large serum volumes were collected on days 21, 35, and 49 (~2 L serum/horse/day) for manufacturing F(ab′)_2_ fragment following the production and quality standards described in the 2015 edition of Chinese Pharmacopoeia,^3^ yielding a total of 1.5 L (26 mg/mL) of therapeutic-grade F(ab′)_2_ that showed a PRNT_50_ at 1/32,000 (Fig. 1b and see Supplementary Table S1 for detailed quality control parameters). The F(ab′)_2_ product was aliquoted (2 mL/vial) and lyophilized for storage.

**Fig. 1:**
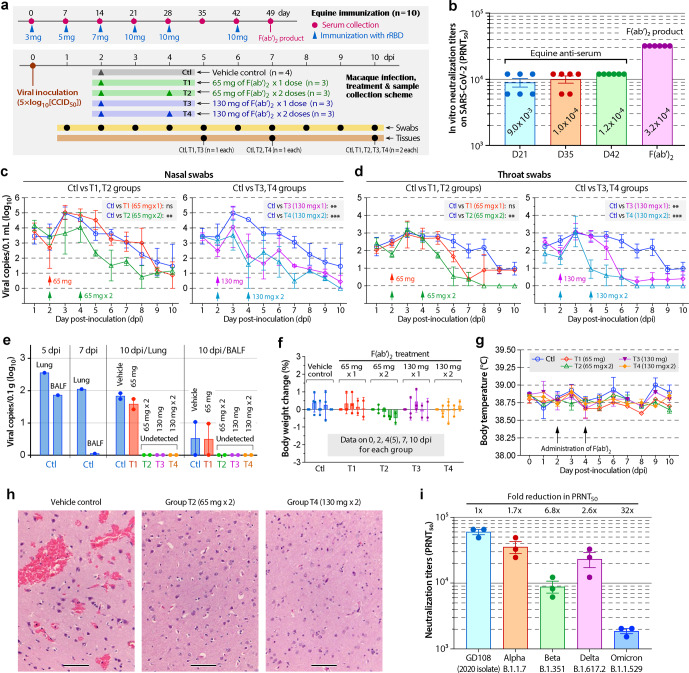
Anti-SARS-CoV-2 efficacy in rhesus macaques of equine F(ab′)_2_ product. **a** Experimental design for the immunization of horses (*n* = 10) with the recombinant receptor-binding domain (rRBD) of the SARS-CoV-2 spike protein and for the treatment of SARS-CoV-2 infections in rhesus macaques with therapeutic F(ab′)_2_ (also see Supplementary Fig. S1 for data on rRBD and Supplementary Table S1 for quality control parameters for F(ab′)_2_ product). **b** In vitro 50% neutralization titers (PRNT_50_) of equine antiserums collected on days 21, 35, and 42 after the first immunization and the prepared F(ab′)_2_ product against the replication of SARS-CoV-2 (KMS-1 isolate). **c**, **d** Viral loads in nasal and throat swabs collected from macaques collected daily from 1 to 10 days post-infection (dpi) as determined by qRT-PCR. Arrows mark the days when F(ab′)_2_ preparations at specified doses were administrated. **P<0.01 and ***P<0.001 between control and specified treatment groups (*n* = 2–4; see detail in Supplementary Table S2) based on two-way ANOVA; ns, no statistical significance. **e** Viral loads determined by qRT-PCR in lung tissues and bronchoalveolar lavage fluids (BALF) collected from macaques sacrificed on 10 dpi. **f** Precent changes of body weights in macaques in the control and treatment groups on 0, 2, 4 (or 5), 7, and 10 dpi. **g** Daily body temperatures in macaques in the control and treatment groups. Arrows mark the days when F(ab′)_2_ preparations were administrated. **h** Representative micrographs of histological sections of brain tissues from the control and treatment groups T2 and T4 showing the resolution of local hemorrhage in macaques receiving F(ab′)_2_ treatments. Also see Supplementary Fig. S2 for a complete set of histological micrographs of tissues from brains, lungs, livers, and spleens. Scale bars for all images, 100 μm. **i** In vitro neutralization titers of the F(ab′)_2_ preparation against the early isolate GD108 and selected variants of concerns (i.e., Alpha, Beta, Delta, and Omicron) assigned with Pango lineages. All bars show standard errors of the means (SEMs)

F(ab′)_2_ product was evaluated for efficacy and safety in 16 rhesus macaques infected with SARS-CoV-2 (KMS-1 isolate; dose at 5 × log_10_[CCID_50_]/animal) via a single nasal spray. Animals were randomly assigned into a control group receiving vehicle (*n* = 4) and four treatment groups (designated as T1 to T4 groups; *n* = 3 each) intravenously receiving F(ab′)_2_ treatments: one dose of 65 mg on day 2 post-infection (dpi) (T1), two doses of 65 mg on 2 and 4 dpi (T2), one dose of 130 mg (T3) and two doses of 130 mg (T4) (Fig. 1a). The combined dosages were equivalent to PRNT_50_ doses at 8 × 10^4^, 1.6 × 10^5^, 1.6 × 10^5^, and 3.2 × 10^5^, respectively, in which PRNT_50_ at 8 × 10^4^ was approximately twofold of the clinical use of 200–500 mL of human-convalescent plasma with typical PRNT_50_ values between 1/40 and 1/80.^4^ Nasal/throat swabs were collected daily for detecting viral loads by qRT-PCR using primers targeting envelope (E) protein. Body weights and body temperatures were measured daily. One animal each from control, T1 and T3 on 5 dpi and one each from control, T2 and T4 groups on 7 dpi were sacrificed. On 10 dpi, the remaining animals were sacrificed (two in each group; also see Supplementary Table S2 for detailed daily specimen numbers). Sacrificed animals were subjected to collection of bronchoalveolar lavage fluids (BALFs) and internal organs for detecting viral loads and/or histology.

All monkeys were successfully infected with SARS-CoV-2. Treatment with F(ab′)_2_ produced a dose-dependent antiviral efficacy. The viral loads were detectable in nasal/throat swabs in all animals on 1 dpi, which peaked at 3 dpi and then start to decline over the time (Fig. 1c, d). In comparison to the control, the decline of nasal/throat viral loads was insignificant in T1 group receiving the lowest dose of F(ab′)_2_, but significant in T2 to T4 groups (*P*<0.01 to *P*<0.001). In tissues collected at 5, 7, and 10 dpi, the viral loads in lungs and BALFs were the highest on 5 dpi but declined at 7 and 10 dpi in the control specimens (Fig. 1e). Note that lungs collected on 5 and 7 dpi were accidentally decomposed, from which virus was detected only from the two control specimens (Supplementary Table S2). On 10 dpi, virus continued to exit in lungs and BALFs in the control and T1 groups, but became undetectable in all other treatment groups (Fig. 1e). All animals showed no obvious mental and dietary changes, and treatment with F(ab′)_2_ had no effect on the animal’s body weights and body temperatures (Fig. 1f, g), supporting the safety of the F(ab′)_2_ product.

In histology of tissues collected on 10 dpi, control monkeys developed pathological changes in brains (local hemorrhage), livers (portal hepatocyte edema), spleen (hemorrhage and enlarged corpuscular germinal center), and lungs (pulmonary hemorrhage, widening of alveolar septa, and varying degrees of lymphocyte infiltration around blood vessels and bronchial tubes) (Supplementary Fig. S2), but no apparent changes in hearts, kidneys, testicles/epididymis, and wombs/ovaries. No improvements were observed on the histopathology in livers, spleens and lungs in all treatment groups. Notably, however, the brain local hemorrhage was fully resolved in T2, T3, and T4 groups (Fig. 1h and Supplementary Fig. S2). In summary, F(ab′)_2_ treatments were safe in monkeys, highly efficacious in reducing the viral loads (T2 to T4), and able to eliminate virus to undetectable levels on 8 dpi in nasal/throat swabs (T4) or 10 dpi in lungs and BALF (T2 to T4) when administrated at reasonably high doses.

Therapeutic-grade equine immunoglobins can be rapidly produced and historically considered as an emergency treatment option in outbreaks (such as during the SARS-CoV-1 outbreak in 2003 in China), and approved in countries like China and United States for producing therapeutic antibodies for human use. There are at least seven FDA-approved equine-derived immune globulin products. The excellent anti-SARS-CoV-2 efficacy demonstrated in a nonhuman primate model here provides strong proof-of-concept data for use of F(ab′)_2_ as an option to treat SARS-CoV-2 infections. In a more recently reported phase 2/3 clinical trial, equine F(ab′)_2_ improved clinical symptoms of hospitalized patients with SARS-CoV-2 pneumonia, particularly those with severe disease.^5^ Because of the high original neutralizing titers, this F(ab′)_2_ product showed lower, but still satisfactory and higher-than-human-convalescent-plasma neutralizing titers on selected SARS-CoV-2 variants (i.e., Alpha, Delta, and Beta variants of concerns) (Fig. 1i). While its neutralizing efficacy on Omicron was unsatisfactory due to the large number of mutations at RBD, new equine F(ab′)_2_ could be quickly produced in response to the emergence of new variants. Another advantage for F(ab′)_2_ is the lack of Fc region, thus avoiding potential Fc-mediated antibody-dependent enhancement (ADE).

In summary, we showed the feasibility to rapidly produce a large quantity of therapeutic equine F(ab′)_2_ with outstanding anti-SARS-CoV-2 efficacy on an early isolate and satisfactory efficacies on selected variants. We also demonstrated the safety and excellent efficacy of the equine F(ab′)_2_ product against SARS-CoV-2 infection in rhesus macaques, which paves the way for its clinical investigation and/or experimental treatment option.

## Supplementary information

SUPPLEMENTAL MATERIAL_clean

## Data Availability

The data that support the findings of this study are available from the lead corresponding author upon reasonable request.
