# Supplementary material for: Rapidly developable therapeutic-grade equine immunoglobulin against the SARS-CoV-2 infection in rhesus macaques
Source: Signal Transduct Target Ther. 2022 Jul 7;7:219. doi: 10.1038/s41392-022-01095-8 (PMC9261890; doi:10.1038/s41392-022-01095-8)
Supplement: Supplementary file 1 — SUPPLEMENTAL MATERIAL_clean [file 41392_2022_1095_MOESM1_ESM.docx]

**Additional Information (Supplementary Materials)**

**Rapidly developable therapeutic-grade equine immunoglobulin against the SARS-CoV-2 infection in rhesus macaques**

Xiaolei Liu^1, #^, Yi Liu^1, #^, Xuemin Jin^1, #^, Zhanlong He^2, #^, Zhen Huang^3, #^, Shumin Sun^4^, Yuwei Gao^5^, Jingyu Li^6^, Qin Ning^7^, Zhongping Xie^2,*^, Ningyi Jin^5,*^, Mingyuan Liu^1,*^

*^1^State Key Laboratory for Zoonotic Diseases, Key Laboratory for Zoonosis Research of the Ministry of Education, Institute of Zoonosis, and College of Veterinary Medicine, Jilin University, Changchun 130062, China; ^2^Institute of Medical Biology, Chinese Academy of Medicine Sciences & Peking Union Medical College, Yunnan Key Laboratory of Vaccine Research and Development on Severe Infectious Diseases, Kunming 650118, China; ^3^Walvax Biotechnology Co., Ltd, Kunming, 650106 China; ^4^College of Animal Science and Technology, Inner Mongolia University for Nationalities, Tongliao 028000 Inner Mongolia, China; ^5^Changchun Veterinary Research Institute, Chinese Academy of Agricultural Sciences, Changchun, China; ^6^Yuxi JOZO Biotechnology co., LTD, Yuxi 653199, China;^7^National Medical Center for Major Public Health Events, Wuhan, China; State Key Laboratory for Zoonotic Diseases, Wuhan, China; Department and Institute of Infectious Disease, Tongji Hospital, Tongji Medical College, Huazhong University of Science and Technology, Wuhan 430000, China.*

*^#^These authors contributed equally: Xiaolei Liu, Yi Liu, Xuemin Jin, Zhanlong He and Zhen Huang.*

**Correspondence: Mingyuan Liu (liumy36@163.com), Ningyi Jin (ningyik@126.com) or Zhongping Xie (xzp218@hotmail.com)*

**This PDF file includes:**

1. Materials and Methods.
2. Figure S1 and S2.
3. Table S1 and S2

**Materials and Methods**

**Ethics declarations**

Horse immunization experiment was approved by the Institutional Animal Care and Use Committee of Jilin University (approval number KT202002069). Macaque experiment was approved by the experimental animal ethics committee of the Institute of medical biology, Chinese Academy of Medical Sciences (approval No. DWSP 202001020 and protocol No. MXMQ202010). All animals were handled in accordance with the relevant governmental regulations in China. All experiments followed approved procedures.

**Animals**

Ten healthy equines aged 4 to 6 years were housed under standard breeding conditions after quarantine inspection and used for the production of antiserums. Sixteen *Rhesus macaques* aged 2 to 3 years were housed in the Primate Experimental Animal Center, Institute of Medical Biology, Chinese Academy of Medical Sciences (Experimental Animal Production License No.: scxk (Dian) k2015-0004). Viral infection and treatment experiments were conducted in the animal biosafety level III (ABSL-3) Laboratory certified by China National Accreditation Service for conformity assessment (CNAS). The experimental work on SARS-CoV-2 virus has been approved by the National Health Commission.

**Virus and host cells**

The SARS-CoV-2 wild type strain (SARS-CoV-2-KMS-1/2020; genome sequence deposited at the GenBank No.: MT226610.1) used in the animal study was isolated from the respiratory mucus of an adult male patient at the Yunnan Hospital of Infectious Diseases in Kunming city in January 2020. SARS-CoV-2 strains for in vitro assays included: 1) a prototypic strain (GD108) and a Beta variant (B.1.351, GDPCC-nCOV84, CSTR.16698.06. NPRC 2.062100001) were kindly provided by the Guangdong CDC; 2) an Alpha variant (B.1.1.7, SARS-CoV-2/C-Tan-BJ202101(B1.1.7), CSTR.1669 8.06. NPRC 2.062100002) was provided by the China CDC; 3) a Delta variant (B.1.617.2, CQ79, CSTR.16698.06. NPRC 6. CCPM-B-V-049-2105-8) was provided by the Chongqing CDC; and 4) an Omicron variant (B.1.1.529 CCPM-B-V049-2112-18) was provided by the Institute of laboratory animal sciences, CAMS&PUMC. The viruses were propagated in Vero cells (ATCC, Manassas, USA) and purified by plaque cloning. Vero cells were cultured in DMEM medium (Corning, NY, USA) containing 5% fetal calf serum (FCS; HyClone, Logan, USA) at 37°C in an CO_2_ (5%) cell culture incubator.^1^

**Evaluation of the binding activity of SARS-CoV-2 rRBD to human angiotensin I converting enzyme 2 (ACE2) receptor by ELISA**

The recombinant protein of the SARS-CoV-2 spike protein receptor binding domain (rRBD; based on GenBank No. 43740568) was purchased from (Bioworld, Atlanta, GA, USA; # NCP0029P). The purity of prepared rRBD was 90.2% as determined by SDS/PAGE. The binding activity between rRBD and recombinant ACE2 (rACE2) (Genscript, Piscataway, NJ, USA, # Z03484) was evaluated by ELISA. Briefly, ELISA plates were precoated with serially diluted rRBD (20 ng/mL to 20 μg/mL) in 0.1 M carbonate-bicarbonate buffer (pH 9.5) at 4 °C overnight. After blocking with 5% BSA in PBST (PBS containing 0.1% Tween-20) for 1 h at 37 °C, rACE2 (2.5 μg/mL) in PBST was added and incubated for 1 h at 37 °C. After four washes, the bound rACE2 was detected using a goat anti-ACE2 antibody (0.5 μg/mL, R&D system) for 2 h at 37 °C, followed by incubation with horseradish peroxidase (HRP)-conjugated rabbit anti-goat IgG antibody (1:5,000, Thermo Fisher Scientific) for 1 h at 37 °C. The reactions were visualized by adding 100 μL substrate 3,3’,5,5’-Tetramethylbenzidine (TMB) (Sigma, St. Louis, MO, USA) and stopped by H_2_SO_4_ (1 N). The optical density at 450 nm (OD_450_) was measured using an ELISA plate reader (Tecan, San Jose, CA).

**Equine immunization**

The recombinant SARS-CoV-2 rRBD protein was used as an immunogen to produce antiserums in horses. The immunogen was emulsified in Freund's complete adjuvant (Sigma-Aldrich, Taufkirchen, Germany; # F588) for first injection or incomplete adjuvant for the subsequent injections at a ratio of 1:1 (v/v). Horses were subcutaneously injected with immunogen at different dorsal positions with emulsified adjuvants on days 0 (3 mg of antigen), 7 (5 mg), 14 (7 mg), 21 (10 mg), 28 (10 mg) and 42 (10 mg). Serum samples were collected from the jugular vein on days 7, 14, 21, 35 and 42 to monitor antibody responses. Large amounts of plasma were collected on day 21, 35 and 49. Pre-immune serums were collected prior to the initial immunization. Serums were stored at -80 ℃.

**Evaluation of the titers of equine antiserums by ELISA**

The 96-well ELISA plates were coated with the recombinant SARS-CoV-2 Fc-S1 protein (Sino Biological, Beijing, China, # 40591-V02H) (2.5 μg/mL; 50 μL/well) overnight at 4 ℃. After three washes with PBST, plates were blocked with 2% defat milk at 37 ℃ for 2 h, and incubated with serially diluted equine serums (1:100, 1:400, 1:1,600, 1:8,000, 1:16,000, 1:32,000 and 1:48,000) in PBST (100 μL/well) at room temperature for 1 h. PBST and irrelevant serums were used as controls. After three washes with PBST, the plates were incubated with HRP-conjugated secondary antibodies (1:2,000) at room temperature for 45 min, followed by four washes in PBST. The plates were developed using TMB reagent and absorbance (OD450) was read as described above.

**Preparation of** **F(ab′)_2_ fragments**

All reagents used in preparation of F(ab′)_2_ fragments were analytical grade. For producing bench-scale pilot product, 1.0 L of equine plasma was mixed with 4.0 L of water and 15 mL of 90% phenol solution in a sterile reactor. The pH was adjusted to 4.3 with 0.1 M HCl (Sigma-Aldrich, # 258148). During agitation, 10 U/L pepsin was added into the mixture, followed by the adjustment of pH to 3.2 with 0.1 M HCl and continuous agitation for 15 min. The sample was stirred at 37 °C, during which the pH was adjusted to 4.2 with sodium hydroxide. Under constant agitation, sodium pyrophosphate decahydrate (final concentration: 12.6 mM), toluene (final concentration: 10 μM) and ammonium sulfate (final concentration: 12% (m/v)) were sequentially added into the mixture, followed by an additional incubation for 1 h at 55°C as described.^2^ The final F(ab′)_2_ product was prepared at Yuxi JOZO Biotechnology Co. according to the 2015 edition of Chinese Pharmacopoeia standards for therapeutic equine immunoglobin. After quality assessment as described below, the final F(ab′)_2_ product (1.5 L at 26 mg/mL) was aliquoted into 52 mg/vial, lyophilized in glass penicillin bottles and stored at -80 °C.

**Quality control assessment of the F(ab′)_2_ preparations**

Protein concentrations were determined by a modified Biuret test by mixing 50 μL samples in 2.5 mL Biuret reagent, followed by incubation at room temperature for 30 min and reading of OD_540_. Standard curves were generated using serially diluted BSA. The pH values were measured with a pH meter equipped with a glass electrode. Sodium chloride was quantified as follows: 1.0 mL of each sample was titrated with 5 mL of 0.1 M silver nitrate, followed by addition 10 mL of 8.0 M nitric acid, heating until the solution was clear, and cooling to room temperature. Pure water (50 mL), ammonium ferric sulfate (8%, 1 mL; as an indicator), and ammonium thiocyanate (0.05 M) were gradually added into the mixture until the solution became consistent light brownish (end point). Osmolarity was assessed cryoscopically with a micro-osmometer. Endotoxin levels were measured by the chromogenic Limulus Amoebocyte Lysate (LAL) assay (Pyrotell, ACC; Massachusetts, USA, # 65003). Lipopolysaccharide was used as positive controls and sterile water was used as a negative control. Sterility test was conducted by filtering product through nitrocellulose membranes (pore size = 0.22 µm), followed by incubation of the membranes in Tryptose Soya Agar for fungi or Sabouraud-glucose Agar for aerobic and anaerobic bacteria for 14 days at 25 ℃ or 35 ℃, respectively. During and at the end of the incubation period, media were microscopically examined for microbial growth. Sterility compliance was dependent on the absence of microbial growth.^3^

**Plaque reduction neutralization assay (PRNT)**

Vero cells were cultured and maintained in MEM medium containing 10% FBS, 100 U/mL penicillin and 100 g/mL streptomycin at 37°C under 5% CO_2_. Prior to the assay, Vero cells (10^5^ cells/mL) were seeded in 24-well plates for incubation overnight. Serially diluted antibodies were mixed with 100 plaque forming units of specified strains of viruses and pre-incubated at 37 °C for 1 h before being added to the cultured cells. After 1 h incubation at 37 °C, supernatants were removed and replaced with 1.2% Avicel solution in DMEM. After 3 days of culture at 37 °C, cell monolayers were fixed with 6% formaldehyde in PBS and stained with crystal violet. Viral plaques were microscopically counted. The EC_50_ values were calculated using a nonlinear regression model (four parameters). Two independent experiments were performed with three technical duplicates.

**Study design: Viral infection and F(ab′)_2_ treatment in macaques**

A total of 16 rhesus macaques (age: 2–3 years, weight: 2–3 kg) were assigned randomly into five groups including one control group (n = 4) and four treatment groups (n = 3 each). All animals were infected with KMS-1 strain of SARS-CoV-2 (10^5^ CCID50/100 µL) intranasally on day 0, and subjected to the following treatment design (also see illustration in Fig. 1a and Table S2): 1) negative control group received no treatment; 2) treatment group 1 (T1) received a single dose of 65 mg of F(ab′)_2_ prepared in 2.5 mL on 2 dpi (day post-inoculation); 3) treatment group 2 (T2) received two doses of 65 mg of F(ab′)_2_ on 2 and 4 dpi; 4) treatment group 3 (T3) received 130 mg of F(ab′)_2_ on 2 dpi; and 5) treatment group 4 (T4) received two doses of 130 mg of F(ab′)_2_ on 2 and 4 dpi. A dose of 65 mg of F(ab′)_2_ was approximately equal to the clinical use of 500 mL of convalescent plasma based on the average neutralizing titer of convalescent plasma at 1:80.^4^

During the course of animal experiment, body temperatures, body weights, general appearance and behaviors, mental state, food intake, respiratory state, fecal properties, genitalia, death and other toxic manifestations were monitored daily. Nasal swabs and pharyngeal swabs were collected daily for detecting the viral loads. Animals were euthanized at 5 dpi (control, T1 and T3, n = 1 each), 7 dpi (control, T2 and T4, n = 1 each) and 10 dpi (all five groups, n = 2 in each group) for collecting main tissues (e.g., brain, lung, heart, liver, kidney, spleen, testis/ovary and epididymis/uterus) for histopathology and detection of viruses. Tissues for light-microscopic examinations were fixed in 10% formalin in PBS, embedded in paraffin, sectioned (4 μm thickness) and stained with haematoxylin and eosin. Representative images were captured and stored in Tagged Image File format. For detection of viral loads, specimen pieces were stored at −80 °C freezers prior to the isolation of RNA. Total RNA was isolated from 100 mg of tissue samples using TRIzol reagent (Tiangen, Beijing, China, # DP424). Note that the specimens collected on 5 and 7 dpi were unfortunately decomposed in an equipment failure during one of the city/laboratory lockdowns. Only specimens from the control groups were rescued for isolation of good quality of total RNA. Viral loads were determined by a TaqMan-based qRT-PCR using TaqMan Fast Virus 1-Step Master Mix kit (Thermo Fisher Scientific, USA) and the following primers and probe: COVID-19 Forward primer: 5’-ACA GGT ACG TTA ATA GTT AAT AGC GT-3’; COVID-19 Reverse: 5’-ATA TTG CAG CAG TAC GCA CAC A-3’; and internal probe: FAM-5’-ACA CTA GCC ATC CTT ACT GCG CTT CG-3'-TAMRA. qRT-PCR reactions were performed using a CFX384 Touch Real-Time PCR Detection System (Bio-Rad, USA). Purified SARS-CoV-2 RNA was used to generate standard curves.

**Statistical analysis**

All the results were expressed as the mean ± standard errors of the means (SEMs). Statistical significances were determined using Student’s *t* test, one-way or two-way analysis of variance (ANOVA) using Prism software (GraphPad, San Diego, CA). *P*-values were expressed as **P*<0.05, ***P*< 0.01 and ****P*<0.001.

**Reference**

1 Xie, T. et al. Three doses of prototypic SARS-CoV-2 inactivated vaccine induce cross-protection against its variants of concern. *Signal. Transduct. Target. Ther.* **7**, 61 (2022).

2 Cunha, L. E. R. et al. Polyclonal F(ab')_2_ fragments of equine antibodies raised against the spike protein neutralize SARS-CoV-2 variants with high potency. *iScience* **24**, 103315 (2021).

3 Leon, G. et al. Development and characterization of two equine formulations towards SARS-CoV-2 proteins for the potential treatment of COVID-19. *Sci. Rep.* **11**, 9825 (2021).

4 Li, L. et al. Effect of convalescent plasma therapy on time to clinical improvement in patients with severe and life-threatening COVID-19: A randomized clinical trial. *JAMA* **324**, 460-470 (2020).

| 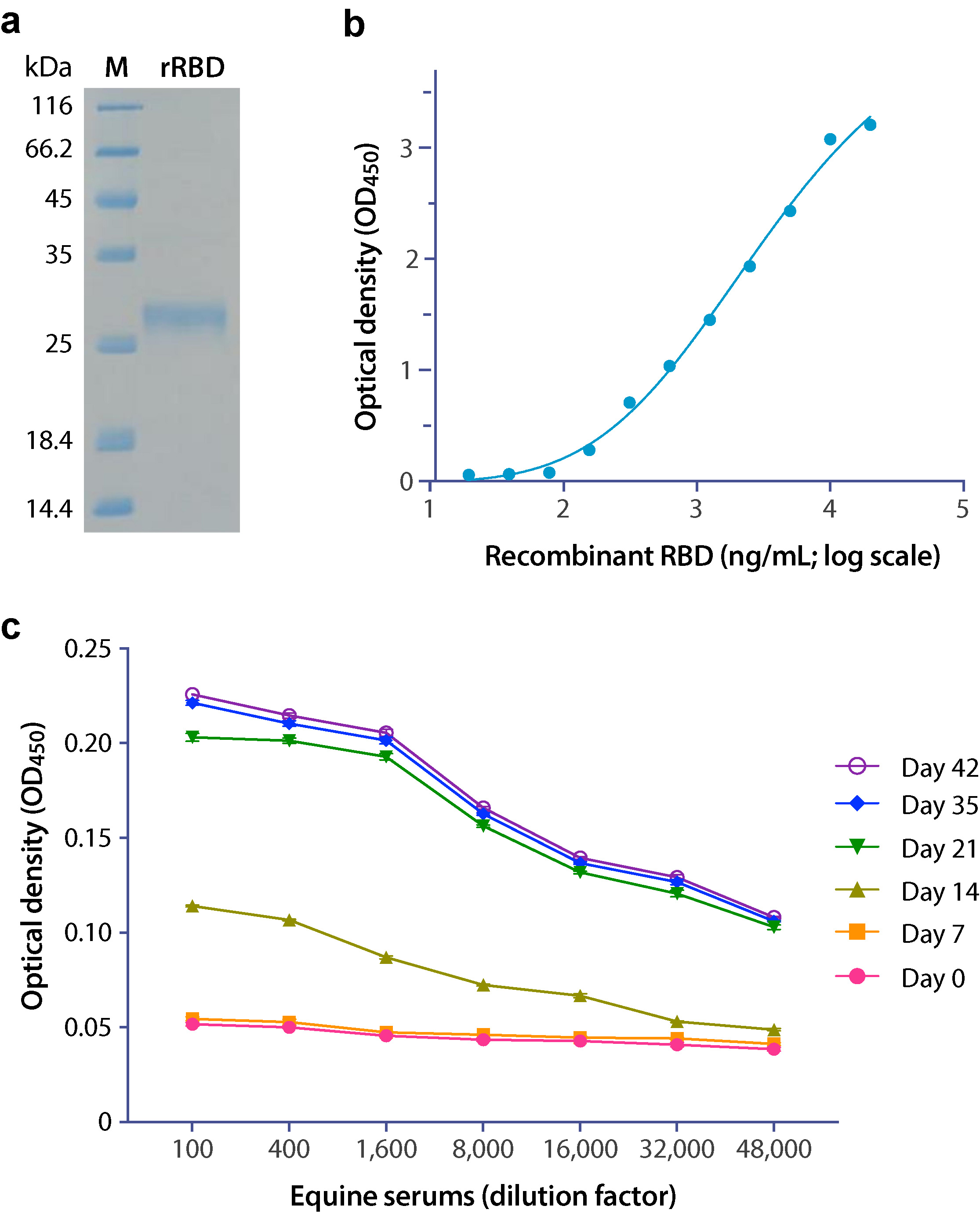 |
| --- |
| **Fig. S1. Binding activity between tag-free recombinant RBD (rRBD) and angiotensin I converting enzyme 2 (ACE2) and the titers of equine antiserums to rRBD.** (a) SDS-PAGE of the tag-free recombinant rRBD stained with Coomassie Brilliant Blue. M, protein marker. (b) The binding activity of rRBD to ACE2 as determined by ELISA. (c) Titers of equine antiserums collected on various days after initial immunization for binding to rRBD as determined by ELISA (days 0, 7 and 14, n = 3; days 21, 35 and 42, n = 10). The data shown were representative of two independent experiments. |

| 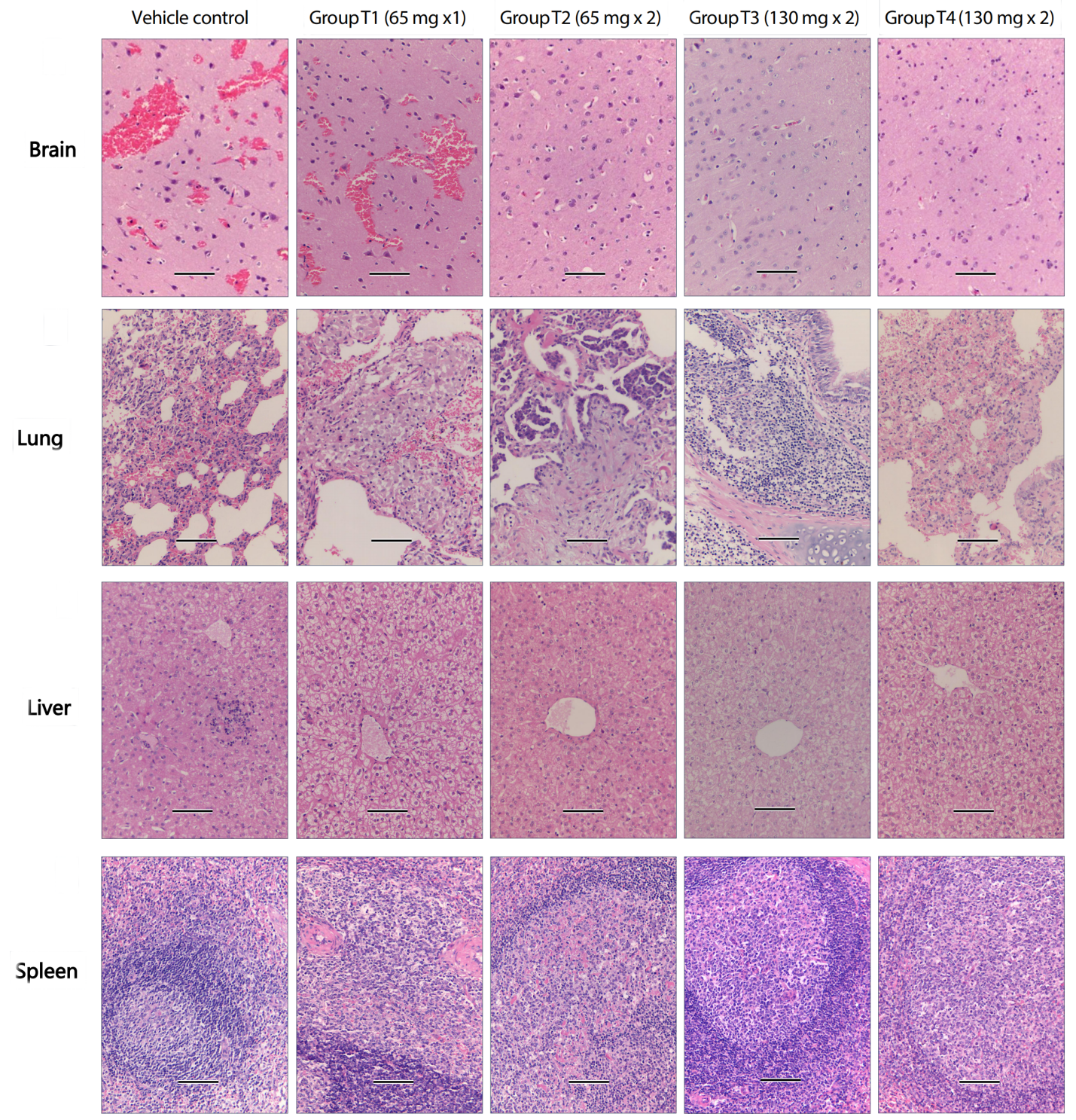 |
| --- |
| **Fig. S2. Histopathology of specimens of brains, lungs, livers and spleens from macaques infected with KMS-1 strain of SARS-CoV-2 on 10 dpi (day post-infection).** Scale bars for all images, 100 μm. |

**Table S1. Quality control assessment of therapeutic grade equine F(ab′)_2_ product**

| **QC parameters** | **Equine F(ab′)_2_ product in this study** | **Chinese Pharmacopoeia Standards^*^** |
| --- | --- | --- |
| F(ab′)_2_ purity (%) | 90.2 | ≥70% |
| Total protein (g/dL) | 20.4 ± 0.06 | < 100 |
| pH | 6.69± 0.03 | 6.0-7.0 |
| Chloride (g/L) | 5.03 ± 0.02 | 7.5-9.5 |
| Ammonium sulfate (g/L) | 0.10 ± 0.00 | < 1 |
| Osmolarity (mOsmol⁄kg) | 328.0 ± 1.2 | ≥240 |
| Limulus amoebocyte lysate (EU/mL) | < 2 | < 25 |
| Sterility | Absence of growth | Absence of growth |

^*^Based on the 2015 edition of the Chinese Pharmacopoeia Standards for therapeutic equine immunoglobin.

| **Table S2**  **Table 2A.** Numbers of specimens in nasal and throat swabs in individual groups during the cause of experiments. Swabs were collected prior to the sacrifice of animals on specified groups and days as shaded in light yellow. | | | | | | | | | | | | | | | | | | | | | | | | | | | | |  |  |
| --- | --- | --- | --- | --- | --- | --- | --- | --- | --- | --- | --- | --- | --- | --- | --- | --- | --- | --- | --- | --- | --- | --- | --- | --- | --- | --- | --- | --- | --- | --- |
| **DPI** | **1 dpi** | **2 dpi** | | | **3 dpi** | | | **4 dpi** | | | **5 dpi** | | | **6 dpi** | | | **7 dpi** | | | **8 dpi** | | | **9 dpi** | | | | | **10 dpi** | | |
| Control (Ctl) | 4 | 4 | | | 4 | | | 4 | | | 4 | | | 3 | | | 3 | | | 2 | | | 2 | | | | | 2 | | |
| Treatment 1 (T1) | 3 | 3 | | | 3 | | | 3 | | | 3 | | | 2 | | | 2 | | | 2 | | | 2 | | | | | 2 | | |
| Treatment 2 (T2) | 3 | 3 | | | 3 | | | 3 | | | 3 | | | 3 | | | 3 | | | 2 | | | 2 | | | | | 2 | | |
| Treatment 3 (T3) | 3 | 3 | | | 3 | | | 3 | | | 3 | | | 2 | | | 2 | | | 2 | | | 2 | | | | | 2 | | |
| Treatment 4 (T4) | 3 | 3 | | | 3 | | | 3 | | | 3 | | | 3 | | | 3 | | | 2 | | | 2 | | | | | 2 | | |
|  |  | | |  | | |  | | |  | | |  | | |  | | |  | | |  | | |  |  | | |  |  |
| **Table 2B.** Numbers of sacrificed animals on specified post-infection days* | | | | | | | | | | | | | | | | | | | | | | | | | | | | |  |  |
| **DPI** | **1 dpi** | | **2 dpi** | | | **3 dpi** | | | **4 dpi** | | | **5 dpi** | | | **6 dpi** | | | **7 dpi** | | | **8 dpi** | | | **9 dpi** | | | **10 dpi** | | |  |
| Control (Ctl) |  | |  | | |  | | |  | | | 1 | | |  | | | 1 | | |  | | |  | | | 2 | | |  |
| Treatment 1 (T1) |  | |  | | |  | | |  | | | 1 | | |  | | |  | | |  | | |  | | | 2 | | |  |
| Treatment 2 (T2) |  | |  | | |  | | |  | | |  | | |  | | | 1 | | |  | | |  | | | 2 | | |  |
| Treatment 3 (T3) |  | |  | | |  | | |  | | | 1 | | |  | | |  | | |  | | |  | | | 2 | | |  |
| Treatment 4 (T4) |  | |  | | |  | | |  | | |  | | |  | | | 1 | | |  | | |  | | | 2 | | |  |
| *Lung specimens collected on 5 and 7 dpi were decomposed in an equipment failure during one of the city/laboratory lockdowns, from which virus was detected only from the control specimens. | | | | | | | | | | | | | | | | | | | | | | | | | | | | |  |  |
